# Magnetoresistive detection of spin waves

**DOI:** 10.1126/sciadv.adx4126

**Published:** 2025-08-15

**Authors:** Quentin Rossi, Daniel Stoeffler, Grégoire De Loubens, Hugo Merbouche, Hicham Majjad, Yves Henry, Igor Ngouagnia, Aurélie Solignac, Matthieu Bailleul

**Affiliations:** ^1^ IPCMS, CNRS, Université de Strasbourg, 67034 Strasbourg, France.; ^2^ SPEC, CEA, CNRS, Université Paris-Saclay, 91190 Gif-sur-Yvette, France.

## Abstract

We explore a detection method for spin waves consisting in integrating a magnetoresistive sensor on a magnonic waveguide. When subjected to the stray magnetic field generated by the spin wave, the relative orientation of the magnetizations of the two magnetic layers in the sensor oscillates in time, resulting in an electrical resistance change according to the so-called giant magnetoresistance effect. Upon application of an appropriate current bias, this variation of resistance translates into a sizable microwave voltage. At the submicrometer scale explored here, this signal is about 50 times larger than the one extracted from conventional inductive measurements of spin waves for comparable detection areas. Moreover, this detection scheme is expected to scale very favorably down to the nanometer size relevant for future magnon-based data processing architectures.

## Teaser

Spin waves are detected very efficiently by coupling them to a magnetoresistive sensor operated in the microwave frequency range.

## INTRODUCTION

Spin waves (or magnons) are low-energy excitations of magnetically ordered materials. In typical ferromagnets, they exist in a range of frequencies (1 GHz to 1 THz) and wavelengths (1 μm to 1 nm) that precisely correspond to relevant time and length scales for modern electronics. This suggests the possibility of using spin waves to develop new architectures for data processing (*1*–*7*), which has motivated the emergence of a field of nanomagnetism called “magnonics” (*8*). In this context, one requires efficient methods for detecting spin waves, in particular convenient ways for converting spin waves into electrical signals of reasonably large amplitude, which is a prerequisite for interfacing magnonics with conventional electronics.

Until now, propagating spin waves have been detected either by magneto-optical imaging [magneto-optical Kerr effect (*9*–*11*) and microfocussed Brillouin light scattering (*12*–*14*)] or by inductive microwave measurements (*15*–*20*). These methods are now reaching their limits in terms of signal sensitivity and spatial resolution, which constitutes a technological bottleneck for the miniaturization of magnonic devices and for the exploration of the fundamental physics of spin waves. Recently, several high-resolution techniques have been developed, such as nitrogen vacancy magnetometry (*21*, *22*) and time-resolved x-ray imaging (*23*, *24*). However, these methods, which require advanced instrumentation, are still limited in terms of versatility and are certainly not directly compatible with a microelectronic environment. As an alternative strategy, we resort to a standard element of spintronics, which is a giant magnetoresistive (GMR) sensor consisting of a multilayer stack comprising two magnetic metal layers separated by a nonmagnetic metal spacer layer (*25*, *26*). Because electrical transport in ferromagnetic metals is spin-polarized, the stack exhibits a different resistance according to the relative orientation of the magnetizations of the two magnetic layers, which constitutes the so-called GMR effect. By a suitable material engineering, this stack can be designed as a high-performance magnetic field sensor (*27*–*29*), already exploited in various applications, including magnetic read heads (*30*), automotive sensors (*31*) or biomedical imaging (*32*).

**Fig. 1.**
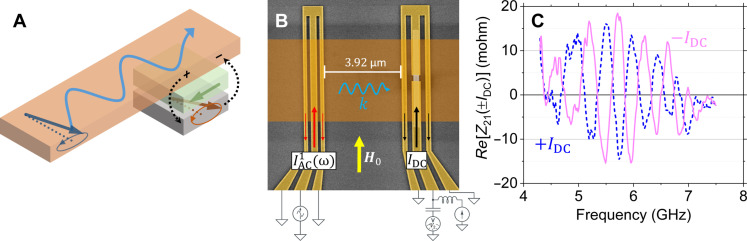
MR detection of spin waves. (**A**) Working principle. In orange, the waveguide in which a spin wave propagates. In gray, the soft (free) layer with its magnetization oscillating around its equilibrium state (dashed line) due to the dipole stray fields generated by the spin wave (dashed arrows). In green, the hard (reference) layer supposed unperturbed by the spin wave. (**B**) Annotated scanning electron microscopy picture of a typical measured sample. Left: The exciting microwave antenna (yellow), connected to port 1 of the vector network analyzer. Right: The sensor in which the gray center part is the MR detection area. The sensor is connected to port 2 of the vector network analyzer. In orange, the Ni_80_Fe_20_ waveguide covering the excitation antenna and the sensor. (**C**) Typical spin-wave signal. The graph shows the real part of the mutual impedance from port 1 to port 2 for a $+I_{\text{DC}}$ and a $-\;I_{\text{DC}}$ bias, dashed blue and solid magenta lines, respectively, with $|I_{\text{DC}}|=1.5$ mA.

In this work, we directly integrate such a spin-valve sensor under a ferromagnetic track that serves as a waveguide for spin waves (Fig. 1A). The sensor is electrically insulated from the track but is located close enough to be coupled to it via stray dipole fields. When a spin wave passes above the sensor, it generates an oscillating dipole field that induces a precession of the magnetization of one of the ferromagnetic layers of the stack (the free layer), the other being pinned magnetically along an orthogonal direction [crossed magnetizations arrangement providing the maximum field sensitivity (*31*)]. This translates in an oscillation of the resistance of the sensor, which, for a given current bias, results in a voltage that can be accessed via suitable microwave measurements. The key advantage of the proposed detection scheme stands in the signal scaling law: the signal of a conventional inductive detection scales as the area of the sensing antenna, whereas the magnetoresistive (MR) signal depends on the available voltage and magnetic susceptibility of the free layer, which can remain very high down to nanometer dimensions (*33*).

## RESULTS

### Device and experimental procedure

The fabricated device is made out of three main parts (Fig. 1B). First, spin waves are excited by an antenna (*15*), which consists of a set of three submicrometer-wide conducting tracks in the coplanar waveguide geometry (left gold structure in Fig. 1B). It is connected to port 1 of a vector network analyzer used to inject a microwave current $I_{\mathrm{AC}}^{1}$ with a frequency ω/2π in the range of 10 MHz to 50 GHz. An oscillating microwave Ørsted stray field is thus generated, which is capable of pumping spin waves with wavelengths in the range of 1 to 2 μm in the nearby ferromagnetic slab.

The latter, made of Permalloy (Ni_80_Fe_20_), serves as a waveguide for spin waves and constitutes the second part (orange track in Fig. 1B). It is 60 μm long, 7 μm wide, and 22 nm thick and is located about 350 nm above the antenna. Its magnetization is saturated along its width using an external static field $H_{0}$ produced by an electromagnet, which corresponds to the so-called Damon-Eshbach configuration of spin-wave propagation (*34*).

The third part of the device is a GMR spin valve optimized for low-frequency sensing applications (*35*, *36*), which consists of the following layers: Si (intrinsic)/SiO_𝑥_(100)/Ta(3)/NiFe(5)/CoFe(2.1)/Cu(2.9)/CoFe(2.1)/Ru(0.85)/CoFe(2)/IrMn(7.5)/Ru(0.4)/Ta(5) where numbers are the nominal thicknesses in nanometer. This stack can be decomposed into three functional elements. (i) The composite NiFe/CoFe free layer (gray in Fig. 1A) is magnetically soft, and its magnetization can easily follow any in-plane magnetic field applied to it. (ii) The CoFe/Ru/CoFe/IrMn part is made magnetically harder taking advantage of the antiferromagnetic coupling through the Ru interlayer. Moreover, by virtue of the exchange bias brought by the antiferromagnetic IrMn layer, a preferred orientation is imprinted using a prior annealing under magnetic field. Overall, this results in a preferred magnetic direction for the first CoFe layer referred to as the reference layer (green arrow in Fig. 1A). (iii) The Cu spacer magnetically decouples the two magnetic parts, while allowing for spin-polarized electrons to flow across it, resulting in an overall resistance change according to the relative orientation of the magnetizations of the reference and free layers.

Using electron-beam lithography and ion-beam etching, the GMR stack is patterned into a 200 nm–by–10 μm strip, with the long axis oriented perpendicular to the annealing field direction. The width of the strip is chosen slightly smaller than a quarter of wavelength, such that the spin-wave stray field can be considered uniform over it. It is also made very long to emulate translation invariance along this direction. Because of the resulting magnetic shape anisotropy, the equilibrium orientation of the free layer (dashed line in Fig. 1A) is orthogonal to that of the reference layer. This strip is contacted to a coplanar waveguide very similar to the excitation antenna, except that it is interrupted over a length of about 600 nm (gray pad on the right of Fig. 1B), which defines the active GMR area. This coplanar waveguide is connected to port 2 of the vector network analyzer. A direct current ( $I_{\mathrm{DC}}$ ) is injected into it using a bias tee. Last, from the measured microwave scattering parameters between the two ports, we extract the mutual impedance from port 1 to port 2

$$Z_{21}\left(\omega \right)=\frac{V_{2}\left(\omega \right)}{I_{\mathrm{AC}}^{1}\left(\omega \right)}$$ (1)

where $V_{2}$ is the microwave voltage at port 2. This procedure is applied first for a high field that serves as a reference and then for the operating field. The measured signal corresponds to the difference between the two datasets, thus removing any direct electromagnetic coupling between the antenna and the sensor.

### Microwave measurements

A typical measured signal is plotted in Fig. 1C, which displays the real part of the mutual impedance as a function of the frequency for an external field of $\mu_{0}H_{0}=20$ mT, an excitation microwave power of P=−15 dBm and bias currents of $I_{\mathrm{DC}}=+\;1.5$ and −1.5 mA (dashed blue and solid magenta, respectively). The oscillatory behavior of the signal is typical of propagating spin-wave spectroscopy (*15*, *37*–*39*). At a given frequency, a magnon of wave vector *k* is excited according to the dispersion relation ω(k) . Upon propagation over a distance *D*, the spin wave acquires a phase delay *kD*, which evolves as a function of frequency, thus generating the oscillation. When switching the polarity of the bias current, one observes an inversion of the measured waveform, which is consistent with an MR origin of the signal (for a given magnetic perturbation, i.e., a given resistance change, the voltage generated is proportional to the current). We observe that this inversion is not perfect (the amplitude of the magenta waveform being slightly larger than that of the blue one). This is attributed to the usual bias-independent contribution of inductive origin, related to the magnetic flux induced by the spin wave in the coplanar waveguide connected to the MR sensor. The measured voltage $V_{2}$ across the sensor can thus be splitted into two contributions

$$V_{2}=V_{2,\text{ind}}+V_{2,\text{GMR}}\left(I_{\mathrm{DC}}\right)$$ (2)

with $V_{2,\text{ind}}$ being the bias-independent inductive voltage (*15*) and $V_{2,\text{GMR}}$ being the GMR voltage that writes

$$\begin{array}{c} V_{2,\text{GMR}}=-I_{\text{DC}}\times \frac{\Delta R_{\text{GMR}}}{2}\times \text{sin}\left(\theta_{0}\right)\text{sin}\left[\text{δθ}\left(\omega \right)\right] \end{array}$$ (3)

Here, $\Delta R_{\text{GMR}}=5\%\times R_{\text{GMR}}$ with $R_{\text{GMR}}=81\;$ ohms, $\theta_{0}$ is the spatially averaged static angle between the in-plane magnetization components of the free and reference layers, and δθ(ω) is the dynamic deviation with respect to this value, which depends on the angular frequency ω. To distinguish these two contributions in the total signal, we extract the sum and the difference of the mutual impedance measured for the two bias polarities, thus defining $\Sigma Z_{21}=\frac{1}{2}\left[Z_{21}\left(-I_{\mathrm{DC}}\right)+Z_{21}\left(+I_{\text{DC}}\right)\right]$ and $\Delta Z_{21}=\frac{1}{2}\left[Z_{21}\left(-I_{\mathrm{DC}}\right)-Z_{21}\left(+I_{\mathrm{DC}}\right)\right]$ , which correspond to the inductive and MR contributions, respectively. From Eq. 3 and assuming $\delta \theta \ll \theta_{0}=\pi /2$ , one can rewrite $\Delta Z_{21}$ as

$$\Delta Z_{21}=\frac{I_{\mathrm{DC}}}{I_{\mathrm{AC}}^{1}}\times \frac{\Delta R_{\text{GMR}}}{2}\times \delta \theta \left(\omega \right)$$ (4)

**Fig. 2.**
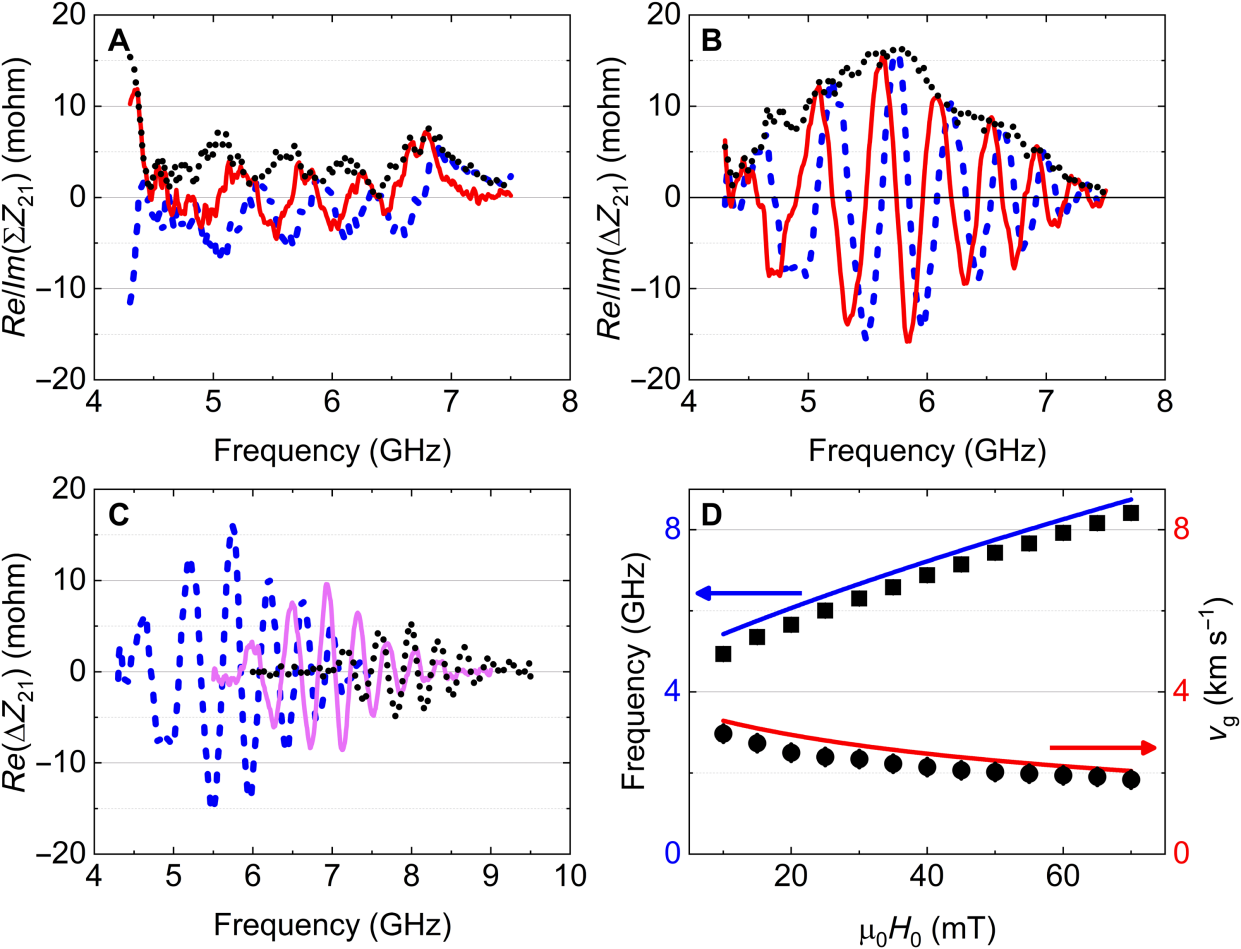
Analysis of the measured mutual inductance. (**A**) Inductive contribution $\Sigma Z_{21}$ to the mutual impedance. Real part (blue dashed curve), imaginary part (red curve), and modulus (black dotted curve). (**B**) MR contribution $\Delta Z_{21}$ to the mutual impedance. Real part (blue dashed curve), imaginary part (red curve), and modulus (black dotted curve). (**C**) Real part of the MR signal measured for several applied external fields, $\mu_{0}H_{0}=20$ , 40, and 60 mT (blue dashed, magenta and black dotted curves, respectively). (**D**) Experimental (black squares and black dots) and theoretical (blue and red lines) estimates of the resonance frequency and group velocity of spin waves (see method of extraction in the Results).

In Fig. 2, we show both the inductive (A) and MR (B) contributions to the total measured signal. These data show that, in the present case, the MR detection exhibits a signal level about five times higher than the usual inductive detection. Noting that the inductive signal is proportional to the antenna’s length covered by the waveguide (*15*) (≈7 μm), whereas the MR signal arises quasi-exclusively from the 600-nm-long detection area, we estimate that the MR signal is indeed 50 times more intense than the inductive one for a given detection volume.

As shown in Fig. 2C, the wave packets shift to higher frequencies upon increasing the field $H_{0}$ as expected from a signal related to magnetic resonance. This is compared to the frequency expected for the spin-wave dispersion of dipole-exchange spin waves in the so-called Damon-Eshbach configuration (*40*) expressed as

$$\begin{array}{c} \begin{array}{c} \omega^{2}=\gamma^{2}\mu_{0}^{2}\left\{H_{0}+M_{s}\left[\Lambda^{2}k^{2}+\left(1-P_{00}\right)-N_{z}\right]\right\}\;\;\\ \;\times \left[H_{0}+M_{s}\left(\Lambda^{2}k^{2}+P_{00}-N_{z}\right)\right] \end{array} \end{array}$$ (5)

where γ/(2π)=29.8 GHz $T^{-1}$ is the gyromagnetic ratio, $H_{0}$ is the applied field, $\mu_{0}$ is the vacuum permeability, $M_{s}=641$ kA $m^{-1}$ is the saturation magnetization, Λ=6.5 nm is the exchange length, **k** is the wave vector of the propagating spin wave, $P_{00}=1-\frac{1-e^{-\mathrm{kT}}}{\mathrm{kT}}$ is the demagnetizing factor associated with the spin wave spatial periodicity, with *T* as the Permalloy slab thickness and $N_{z}=T/\left(W+T\right)$ as the demagnetizing factor accounting for its width *W*. The blue line in Fig. 2D shows the corresponding estimated values of frequency, using magnetic parameters extracted from ferromagnetic resonance (*39*, *41*) and superconducting quantum interference device (SQUID) magnetometry (*42*) of a Permalloy film deposited in the same run (see fig. S2) and a wave vector k≈4 rad μm^−1^ corresponding to the maximum excitation efficiency of our antenna (*15*, *43*).

The measured waveforms in Fig. 2C display a periodicity δf that also depends on the magnitude of the applied field, decreasing from 570 MHz for a field of 10 mT down to 350 MHz for 70 mT. This period is to be identified as the inverse of the delay time τ of spin waves (*37*, *38*) and is therefore directly related to their group velocity $v_{g,\text{exp}}=D/\tau$ , where *D* is the effective distance between the exciting antenna and the GMR sensor. Figure 2D shows the group velocity estimated using the center-to-center distance *D* = 5.2 μm between the excitation antenna and the GMR sensor. The red curve shows the theoretical group velocity obtained by differentiating the spin-wave dispersion (Eq. 5) with respect to the wave vector. One obtains an overall good agreement, which, together with the agreement of the resonance frequency, confirms that the measured waveform corresponds to the propagation of Damon-Eshbach spin waves in the Permalloy slab (see also fig. S7, which shows a clear decay of the MR signal amplitude with increasing distance *D* and an associated increase in the time delay).

### Micromagnetic simulations

**Fig. 3.**
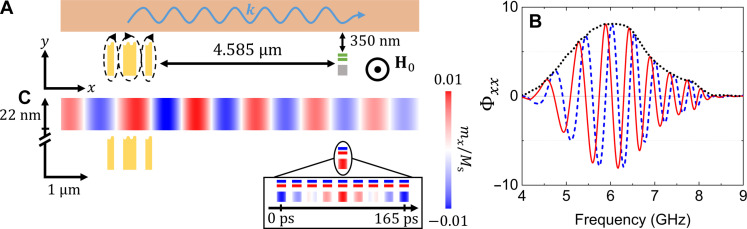
Micromagnetic simulation of the system. (**A**) Cross-sectional sketch of the layout of the simulation containing the exciting antenna (yellow structure) represented with its Ørsted field, the Permalloy slab (orange) in which a spin wave propagates at a wave vector k , and the GMR stack (green and gray). (**B**) Real part (blue dashed curve), imaginary part (red curve), and modulus (black dotted curve) of the response function $\Phi_{\mathrm{xx}}$ relating the oscillation of the free layer to the excitation provided by the microwave antenna. This function provides a numerical equivalent of the transmission spectrum measured in the device. Magnetic field is $\mu_{0}H_{0}=20$ mT. Other magnetic parameters and dimensions are given in Materials and Methods. (**C**) Visualization of simulated spin wave and free-layer oscillations for a microwave frequency of 6.06 GHz [corresponding to the maximum of the modulus in (B)]. The color map shows a snapshot of the normalized in-plane component of the dynamic magnetization $m_{x}$ . For easier visualization, the dynamic magnetization in the GMR stack has been multiplied by a factor of 5. Note the interrupted *y* scale allowing one to visualize both the magnonic waveguide and the GMR stack despite their large vertical separation.

To understand the magnitude of the measured signal and visualize the coupling of the spin wave to the sensor, we performed numerical simulations using the MuMax^3^ micromagnetic simulation software (*44*). The simulation layout is sketched in Fig. 3A. It is designed as a numerical twin of the measured device and contains the same three parts, namely, the antenna excitation field, the magnonic waveguide, and the patterned GMR stack (yellow, orange, and green/gray rectangles in Fig. 3A, respectively). The main simplification is that we conducted a quasi–two-dimensional simulation, emulating translation invariance in the *z* direction using so-called periodic boundary conditions. The magnetic parameters of the Permalloy slab are the ones determined experimentally (see above). The magnetic parameters for the GMR stack correspond mainly to textbook values.

To compute the overall response of the system, we first discretize it in finite difference cells with a size of 2.5 nm by 1 nm by 5 nm. The initial magnetic state is obtained in the presence of the Ørsted field generated by a small quasi-static current flowing in the excitation antenna (see figs. S4 and S5). Then, this field is suppressed abruptly, and the subsequent down-ringing evolution of the magnetic system is computed according to the Landau-Lifshitz-Gilbert equation of motion, accounting for all magnetic interactions acting in the system (in particular, the exchange interaction between neighboring cells and the dipole interaction acting between distant cells). The response function is defined as $\Phi_{\mathrm{xx}}\left(\omega \right)=<{\tilde{m}}_{x}\left(\omega \right){>}_{\text{FL}}/{\tilde{h}}_{x}^{\text{exc}}\left(\omega \right)$ where ∼ stands for the time Fourier transform, $m_{x}$ is the *x* component of the time-varying magnetization, $<\cdot {>}_{\text{FL}}$ is a spatial average over the free layer volume, $h_{x}^{\mathrm{exc}}$ is the *x* component of the time-varying Ørsted field in the magnonic waveguide above the center of the antenna, and *x* stands for the in-plane component along the direction of propagation of the spin wave. Figure 2B shows the real and imaginary parts of this response function calculated for a field of 20 mT. One can clearly identify an oscillating waveform similar to that in Fig. 2B.

In a second step, we replace the stepwise varying excitation by a sinusoidal one corresponding to a microwave current amplitude of $I_{\mathrm{AC}}^{1}=0.46$ mA, choosing the frequency *f* = 6.06 GHz for which the response function was found to be maximum. Figure 3C shows a typical dynamic magnetization map in such a continuous wave propagation regime. One recognizes the propagating spin wave (blue and red alternating patterns) and the attenuation of its amplitude along the length of the waveguide. The dynamics of the magnetization generated in the free layer is also clearly visible. To illustrate this dynamics, the inset in Fig. 3C shows a time sequence of the magnetization distribution in the GMR sensor over a full period of oscillation where one recognizes an oscillation around the equilibrium position (see movie S1 for an animated view). From such a simulation, we calculate the spatially averaged angle between the free layer and reference layer in-plane magnetizations that oscillates in a range of ±δθ=0.07° around a mean value of $\theta_{0}=83.28{}^{\circ}$. Injecting the magnitude of this oscillation in Eq. 4, one obtains a simulated mutual impedance with a magnitude of 8 mohms, to be compared with the measured value of 16 mohms, which corroborates our numerical approach given the approximate modeling of the geometry.

From this simulation, we infer that the free-layer magnetization oscillation is comparable in magnitude to that in the magnonic waveguide above it ( $m_{x}^{\text{FL}}/M_{s}^{\text{FL}}\approx 9\times 10^{-4}$ versus $m_{x}^{\text{SW}}/M_{s}\approx 3.3\times 10^{-3}$ ). This can be understood as follows. The dipole field generated is of order $m_{x}^{\text{SW}}G_{\text{dip}}$ , where $G_{\text{dip}}\approx e^{-\mathrm{ks}}\times \mathrm{kT}/2$ is a dipole mutual demagnetizing factor (*45*), with *s* as the vertical spacing between the waveguide and the sensor. The quasi-static magnetic susceptibility of the free layer is of the order $\chi_{\mathrm{xx}}={\left(H_{0}/M_{s}^{\text{FL}}+t/w\right)}^{-1}$ , with *w* as the width of the GMR sensor and *t* as the thickness of the free layer. In the low-field limit of interest here, one obtains a ratio $m_{x}^{\text{FL}}/m_{x}^{\text{SW}}=G_{\text{dip}}\chi_{\mathrm{xx}}\approx 0.37$ , which is comparable to the simulated one. That is, the smallness of the stray field is compensated by the large sensitivity of the sensor.

## DISCUSSION

### Scaling law

We can now compare the magnitude of the signals expected for an MR sensor and an inductive antenna with the same lateral dimensions. For a given oscillating magnetization angle δθ and in the low-field and low-frequency operation of the MR sensor described above, the generated voltages are $V_{\text{MR}}=I_{\mathrm{DC}}\Delta RG_{\text{dip}}\delta \theta \left(M_{s}/M_{s}^{\text{FL}}\right)\left(w/t\right)$ and $V_{\text{ind}}=\mu_{0}M_{s}\omega G_{\text{ind}}\delta \theta \text{lw}$ , respectively, where *l* is the length of the sensor, $G_{\text{dip}}$ is the dipole coupling factor given above, and $G_{\text{ind}}$ is an inductive coupling factor, which is of comparable magnitude. From these expressions, it is clear that upon scaling down devices, the inductive signal decreases directly with antenna length *l*, while the MR voltage can remain constant with a suitable design. Assuming $M_{s}=M_{s}^{\text{FL}}$ and $G_{\text{dip}}/G_{\text{ind}}=1$ , one gets $V_{\text{MR}}/V_{\text{ind}}=I_{\mathrm{DC}}\Delta R/\left(\mu_{0}M_{s}\omega \text{tl}\right)$ . For a typical frequency of 10 GHz and a magnetization of 1 T, one gets $\mu_{0}M_{s}\omega \approx 10^{11}$ V $m^{-2}$ . Then, with an easily achievable GMR voltage ΔR×I of the order of 10 mV, MR detection becomes more favorable as soon as $l\times t<10^{-13}$ $m^{2}$ . For a 10-nm-thick free layer, this corresponds to a length of the order of 10 μm (see the measurements on a 10-μm scale version of our device in fig. S6, for which we extract a ratio of order unity). For modern MRAM technologies involving ultrasmall magnetic tunnel junctions with large MR ratios (*33*) (*t* = 1 nm, *l* = 10 nm, and ΔR×I=100 mV), the ratio $V_{\text{MR}}/V_{\text{ind}}$ would reach up to $10^{5}$.

### Perspectives

In this work, we have shown that the MR detection of spin waves is possible and that the signal extracted is much larger than the one obtained by the standard inductive technique when working in the submicrometer range. Further improvements of the technique would include the replacement of the GMR sensor with a tunnel magnetoresistance sensor, providing a much higher MR ratio, and the customization of the dimensions and composition of the magnetic free layer to adapt it to the system of interest. A clear price to pay is the increased complexity of the sensor, which displays its own magnetic response, likely to convolute with the spin wave one. However, such a convolution can be treated using models and simulations already well mastered in the magnonic and magnetic sensing communities.

Our result paves the way to design smaller and more efficient spin wave–based devices. This opens highly promising perspectives for fundamental magnonics, enabling study of spin waves in the sub–100-nm range, in the thermal fluctuation regime or in the quantum regime. From a technological point of view, an efficient conversion from nanoscale spin wave to electrical voltage will greatly facilitate the interfacing of magnonics with microelectronics and make possible the implementation of magnonic architectures for advanced computing schemes.

## MATERIALS AND METHODS

### Nanofabrication procedure

The device was fabricated in the STnano nanofabrication platform at institut de physique et chimie des matériaux de Strasbourg. We start with a GMR stack (*35*, *36*) that is grown by magnetron sputtering on thermally oxidized intrinsic silicon wafers using a Singulus production-grade physical vapor deposition facility at service de physique de l’état condensé. After sputtering of the GMR stack, the free layer coercivity is 2.4 Oe, the offset is 6 Oe, and the MR is 7.24%. To pattern the sample, we first perform an electron beam (E-beam) lithography of 10 μm–by–200 nm elements on the full-film GMR stack. In a second step, we evaporate Al(25 nm) and proceed with a lift-off to produce a hard mask for etching. Then, we perform an ion beam etching of the GMR stack. Once etched, we proceed with the E-beam lithography, evaporation, and lift-off of Ti(5 nm)/Au(50 nm) to pattern the submicrometer parts of the microwave antenna and the GMR sensor connectors. Using laser lithography, followed by the evaporation and lift-off of Ti(5 nm)/Au(50 nm), we then pattern the millimeter-scale connection pads of the two coplanar waveguide. Then, we electrically insulate the gold structures by spin coating and hard baking AZ 1505 photoresist (500-nm nominal thickness). Last, using laser lithography, evaporation of Ti(5 nm)/Ni_80_Fe_20_ (20 nm)/Ti(5 nm), and lift-off, we create the Permalloy slab on top of the hard-baked AZ 1505.

### Microwave measurements

After nanofabrication, a sample with a size of 16 mm by 14 mm containing six functional devices is cut and placed in a homemade microwave probe station equipped with an electromagnet with a gap of 10 mm. Each device contains a pair of contact pads of ground-signal-ground geometry, which can be contacted with a pair of microwave probes, GGB Picroprobe 50A with 150-μm pitch. The two probes are connected to the two ports of a Vector Network Analyzer Keysight PNAE8364B via phase-stable microwave cables (port 1 connected to the antenna and port 2 connected to the GMR sensor; see Fig. 1B). The internal bias tee of port 2 is connected to a Keithley 2400 source meter to inject the dc current in the GMR. The microwave power is set to −15 dBm. Each device has an input impedance in the range of 200 to 300 ohms. Before measurements, a suitable short-open-load-through microwave calibration procedure is performed using a GGB Picroprobe CS-5 calibration substrate. An additional port extension is included to account for the electric delay induced by the microwave propagation from the contact pads to the antenna/GMR regions.

Spin-wave measurement procedure is as follows. First, for reference, a large external field of about 200 mT is applied via the electromagnet, and then the calibrated and corrected S parameters are measured by the vector network analyzer. In a second time, the applied field is reduced to the measurement value, a positive dc bias current is established, and the S parameters are measured. This measurement is repeated for an opposite value of the dc bias current, and the measurements are repeated several times to improve the signal-to-noise ratio. Then, the measured S parameters are converted to an impedance matrix (*15*), and the impedance matrix at the high-field reference is subtracted from the impedance matrix at the measurement field, providing the mutual impedance reported in Fig. 1C. Because spin-wave resonances fall far above the frequency range of interest for the high reference magnetic field used, this procedure allows for electromagnetic background subtraction. To extract the spin-wave delay time τ (from which the group velocity of Fig. 2D is deduced), the MR contribution to the mutual impedance is Fourier transformed, and the time for which the resulting time-trace has maximum amplitude is determined.

### Micromagnetic simulations

A numerical twin of the device was developed using the micromagnetic simulation software MuMax^3^ (*44*). The simulation layout is sketched in Fig. 2A. This is discretized into 8192 by 516 by 1 elementary cuboid cells with dimensions of 2.5 nm by 1 nm by 5 nm. These dimensions are smaller than or comparable to the exchange length in the magnetic materials of interest ( $\Lambda =\sqrt{2A_{\text{ex}}/\mu_{0}M_{s}^{2}}$ , with $A_{\text{ex}}$ as the exchange stiffness constant, which is of order of 5 and 3.5 nm for Permalloy and CoFe, respectively), which ensures that the magnetization can be considered uniform over each cell. To emulate a 10-μm extension along the *z* direction, we use the periodic boundary condition feature of MuMax^3^ with 1000 repeats on each side. To model the GMR stack, nonferromagnetic layers are replaced with vacuum, and the magnetic couplings they induce are introduced as an additional magnetic energy contribution acting in adjacent ferromagnetic cells (see fig. S3).

## Supplementary Materials

### The PDF file includes:

Supplementary Text Figs. S1 to S7 Legend for movie S1 References
