# Supplementary material for: Magnetoresistive detection of spin waves
Source: Sci Adv. 2025 Aug 15;11(33):eadx4126. doi: 10.1126/sciadv.adx4126 (PMC12356233; doi:10.1126/sciadv.adx4126)
Supplement: Supplementary file 1 — Supplementary Text Figs. S1 to S7 Legend for movie S1 References [file sciadv.adx4126_sm.pdf]

Science Advances
AAAS

Supplementary Materials for

Magnetoresistive detection of spin waves

Quentin Rossi *et al.*

Corresponding author: Quentin Rossi, quentin.rossi@ipcms.unistra.fr;
Matthieu Bailleul, matthieu.bailleul@ipcms.unistra.fr

Sci. Adv. **11**, eadx4126 (2025)
DOI: 10.1126/sciadv.adx4126

The PDF file includes:

Supplementary Text
Figs. S1 to S7
Legend for movie S1
References

Other Supplementary Material for this manuscript includes the following:

Movie S1

Supplementary Text

GMR characterization. A second device was fabricated to specifically characterize the GMR stack (Fig. S1 inset). This device consists of a GMR strip with dimensions identical to the propagating spin-wave sensor (10 µm × 200 nm). It is contacted with four gold tracks, enabling a four-probes measurement process. The device is then placed in an electromagnet that produces an in-plane static field H_0 perpendicular to the length of the sensor. By varying the applied field and measuring the resulting voltage under current, we extract the resistance of the sensor as a function of the applied field (Fig. S1). In Fig. S1, we observe a high-resistance state R_{AP} that we identify as the antiparallel state between the free layer and the reference layer and a low-resistance state R_{P} that we identify as the parallel state between the free layer and the reference layer. The magneto-resistive ratio MR of the sensor is defined as :

$$\mathrm{MR} = \frac{R_{\mathrm{AP}} - R_{\mathrm{P}}}{R_{\mathrm{AP}}} \approx 0.05. \tag{S1}$$

From these data, we also deduce the sheet resistance $R_{\square}$ of the sensor, taking the measured resistance at zero field divided by the number of squares that make up the surface of the sensor between the voltage contacts. Here $R_{\square} = 27$ ohms.

FMR on Permalloy film. During the evaporation of Ti(5 nm)/$Ni_{80}Fe_{20}$(20 nm)/Ti(5 nm) in the nanofabrication process, four 2×2 mm^2 Permalloy films were also fabricated to extract their magnetic parameters. Using the flip-chip method, they were installed on top of a coplanar waveguide, allowing for broadband ferromagnetic resonance (FMR) characterization (*16*). The coplanar waveguide is placed in between two poles of an electromagnet. These poles are equipped with two modulation coils allowing for field modulated measurements (*47*). The coplanar waveguide is connected to a microwave signal generator on one side and to a microwave diode and a lock-in amplifier on the other side. By performing field sweeps at a given frequency, we extract the resonance line of the Permalloy film, as shown in Fig. S2A. The resonance line is fitted using the derivative of a Lorentzian function, enabling the extraction of both the resonance field and the line-width. The process is repeated for several frequencies. Frequency vs. resonance field and line-width vs. frequency are plotted in Fig. S2B and C, respectively. The frequency vs field data is fitted to a

Kittel-like function (*42*)

$$f = \frac{\gamma\mu_0}{2\pi}\sqrt{(H_0 + H_K)(H_0 + M_{\text{eff}})} \tag{S2}$$

with $\gamma = 187.5 \pm 0.2$ rad GHz T^{-1}, $M_{\text{eff}} = 641 \pm 2$ kA m^{-1} $\approx M_s$ for Permalloy and $H_K = 250 \pm 46$ A m^{-1} a small residual in-plane anisotropy contribution. In Fig. S2C, we have represented the full width at half maximum δH (FWHM) of the resonance peak as a function of the frequency. The FWHM is fitted to the following expression

$$\delta H = \frac{4\pi\alpha f}{\gamma\mu_0} + \delta H_0, \tag{S3}$$

which provides us $\alpha = 0.00907 \pm 0.00005$ and $\mu_0\delta H_0 = 1.22 \pm 0.03$ mT. The effective magnetization of the film is similar to the saturation magnetization ($M_s = 655.2 \pm 0.6$ kA m^{-1}) deduced from a SQUID measurement; both values being about 20% smaller than the tabulated value for permalloy. We attribute this deviation to the deposition of the film on a hard-baked photo-resist spacer, likely to promote some roughness and reduced density. The damping parameter α is also higher than the tabulated value for permalloy, which can again be attributed to the roughness of the film. Finally, the so-called inhomogeneous broadening δH_0 remains small.

Micromagnetic simulations. *The GMR stack.* The synthetic antiferromagnet (SAF) consists of two ferromagnetic CoFe layers separated by a Ru (non-magnetic) spacer of thickness such that the Ruderman-Kittel-Kasuya-Yosida (RKKY) coupling between them promotes an antiferromagnetic alignment between the two CoFe layers. The RKKY coupling constant is $J_{\text{RKKY}} = -0.8$ mJ m^{-2}. The top CoFe layer is subjected to a bias field produced by the exchange coupling with the IrMn antiferromagnet (AF). The corresponding coupling constant is $J_{\text{AF/SAF}} = 0.1$ mJ m^{-2}. The bottom CoFe layer is the reference layer.

The free layer consists of a stack of CoFe and NiFe. In an ideal case, the SAF and the free layer should be completely uncoupled. However, due to the roughness of the Cu spacer, an orange-peel coupling remains. This is almost compensated by a small RKKY coupling between the SAF and the free layer. The remaining coupling constant is $J_{\text{FL/SAF}} = 0.005$ mJ m^{-2}.

The magnetic parameters for the two ferromagnetic materials in the GMR stack are the following: $\gamma_{\text{CoFe}} = 189.12$ rad GHz T^{-1}, $M_{s,\text{CoFe}} = 1400$ kA m^{-1}, $A_{\text{ex,CoFe}} = 15$ pJ m^{-1}, $\alpha_{\text{CoFe}} = 0.01$ for CoFe and $\gamma_{\text{NiFe}} = 189.12$ rad GHz T^{-1}, $M_{s,\text{NiFe}} = 800$ kA m^{-1}, $A_{\text{ex,NiFe}} = 11$ pJ m^{-1}, $\alpha_{\text{NiFe}} = 0.01$

for NiFe. When the simulated GMR stack reaches its equilibrium state under an applied field $\mu_0 H_0 = 20$ mT along the z direction (strip length), the angle between the free layer and the reference layer is 83.28 °. We attribute the deviation from the ideal 90 ° value to a small rotation of the magnetization of the reference layer due to the applied field, and to the small residual magnetic coupling between the free layer and the reference layer.

The microwave antenna. To introduce the antenna into the simulation, we calculate its Ørsted field using the analytical expression of the magnetic field generated by an electrical current flowing uniformly across a a lead with rectangular section (*44*). The resulting x profile of Ørsted field at a height of 350 nm (which corresponds to the location of the Permalloy slab) is shown in Fig. S5A. Due to its spatial periodicity, the antenna acts as a wavelength filter for the excitation. Calculating the spatial Fourier transform of the Ørsted field provides the spectral excitation distribution of the antenna. In Fig. S5B, such a Fourier transform is plotted for a spacing of 350 nm. From Fig. S5B we deduce that the main spin-wave excitation in the permalloy slab occurs at a wave-vector of 4 rad μm^{-1}.

The waveguide. The waveguide consists of a Permalloy slab that is 40 µm along x, 22 nm thick (along y) and 5 µm wide. We use gradually increasing damping values at the $+x$ and $-x$ extremities of the Permalloy slab to avoid spin-wave reflections. The values of gyromagnetic ratio, saturation magnetization and exchange length are the ones indicated in the main text. We use a Gilbert damping $\alpha = 0.009$, as deduced from the ferromagnetic resonance characterization of the Permalloy film (see Supplementary Text).

Stray field estimates. To estimate the microwave magnetic stray field components relevant for both inductive and magneto-resistive coupling, we proceed as follows: For the parameters investigated here ($T \ll \lambda$, $H_0 \ll M_s$), the magnetic precession in the spin-wave is strongly elliptical, with an in-plane component much larger than the out-of-plane one ($|m_x| \gg |m_y|$). For a given spin-wave wave-vector k, the spatial harmonic dependence of the in-plane component ($m_x = m_0 \exp(ikx)$) generates spatially harmonic magnetic volume pseudo-charges in the film ($\rho = -ikm_0 \exp(ikx)$), which, in turn, generate a spatial harmonic magnetic stray field outside of the film. For an estimate of the two components x and y of this stray field, we resort to the plane-wave dynamic demagnetizing factors reported in Ref. (*46*). At a distance $y = s$ larger than the film thickness T, one obtains: $h_x = n_{xx}(k,s)m_x$ and $h_y = -i\,\mathrm{sgn}(k)n_{xx}(k,s)m_x$ (Eq. (32) in Ref. (*46*)) where

$n_{xx}(k,s) = 2\sinh^2(kT/2)e^{-|ks|}/(|k|T) \simeq e^{-|ks|} \times |k|T/2$ (Eq. (26) in Ref. (*46*)). The component acting on the GMR free layer is h_x and the one generating magnetic flux through our elongated planar antenna is h_y, which allows us to identify our coupling factors G_{dip} and G_{ind} from the expressions above, from which we deduce $|G_{\mathrm{dip}}| = |G_{\mathrm{ind}}|$.

Measurements on a 10 µm scale version. Fig. S6A shows a 10 µm scale version of the nanometer-scale device presented in the main text. It consists of a 13 µm-wide 150 µm-long microwave antenna and a 4×12 µm^2 GMR sensor connected to a coplanar waveguide. The device is capped with 100 nm of SiO_2. The 30 nm thick Permalloy film that serves as a waveguide for spin-waves is evaporated on a Si/SiO_2 substrate and is cut in 1.5×2 mm^2 pieces. Then, it is placed on the device using the flip-chip method. The sample is measured using the same microwave measurement procedure as described above. Figs. S6A and B show the inductive and magneto-resistive signals extracted for a field of 30 mT and a bias of 3 mA. As expected, the ratio is less favorable than at the sub-micrometer scale. More precisely, the peak to peak amplitude of the MR signal is about 13 mohms, while that of the inductive signal is about 293 mohms. Accounting for the sensor length in the two cases (12 µm long GMR section vs 150 µm long coplanar waveguide), we obtain a MR/inductive ratio of the order of 1 in that case.

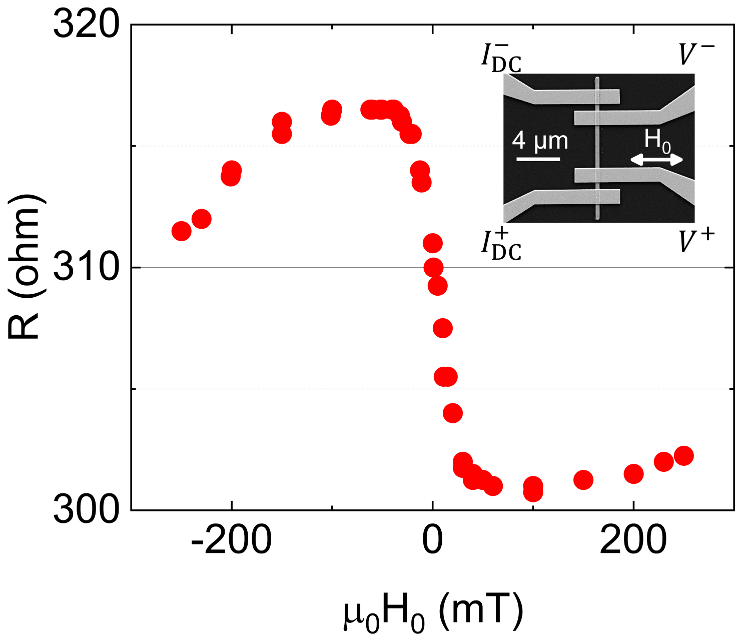

Figure S1: **Four probes measurement of the magneto-resistance.** Red dots represent the resistance R of a GMR strip as function of the magnitude of the external field H_0. Both field and reference layer orientation are perpendicular to the strip. The inset is an annotated scanning electron microscopy picture of the measured device with the respective functions of the connecting pads.

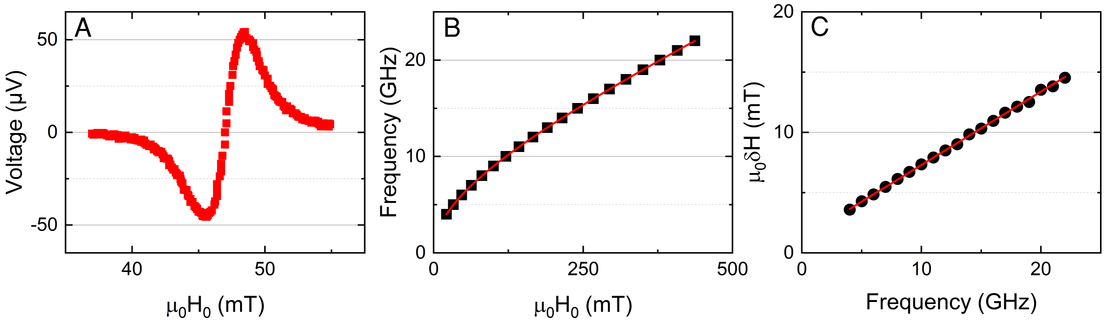

Figure S2: **Ferromagnetic resonance measurements on a Py film.** A Resonance line at a frequency $f = 6$ GHz. B Measured resonance frequency as a function of the applied field (black squares) fitted with Kittel's law (red line). C Linewidth (FWHM) as a function of the frequency fitted with a linear function (red line). This Permalloy film was deposited in the same run as the slab for spin-wave propagation.

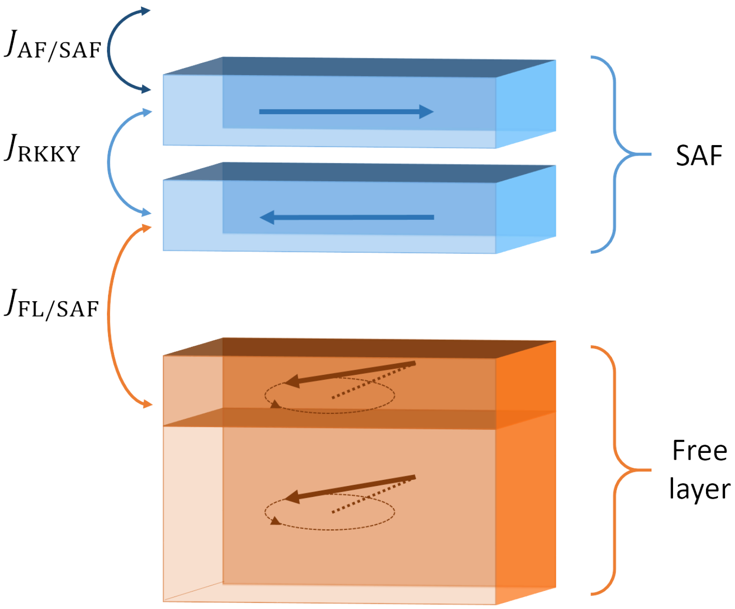

Figure S3: Schematic of the model of GMR stack used in the simulation. The Synthetic AntiFerromagnet (blue) and the respective magnetizations of the CoFe layers are represented as well as the free layer (orange) with the precessing magnetizations of the CoFe (top) and NiFe (bottom) layers. From top to bottom : CoFe(2 nm)/vacuum(1 nm)/CoFe(2 nm, reference layer)/vacuum(3 nm)/CoFe(2 nm)/NiFe(5 nm).

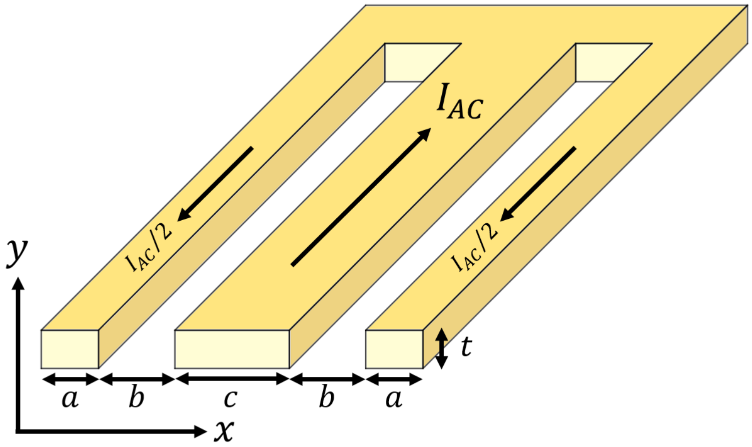

Figure S4: Scheme of the microwave antenna geometry used to calculate the excitation Ørsted field with a = 150 nm, b = 200 nm, c = 300 nm and t = 50 nm.

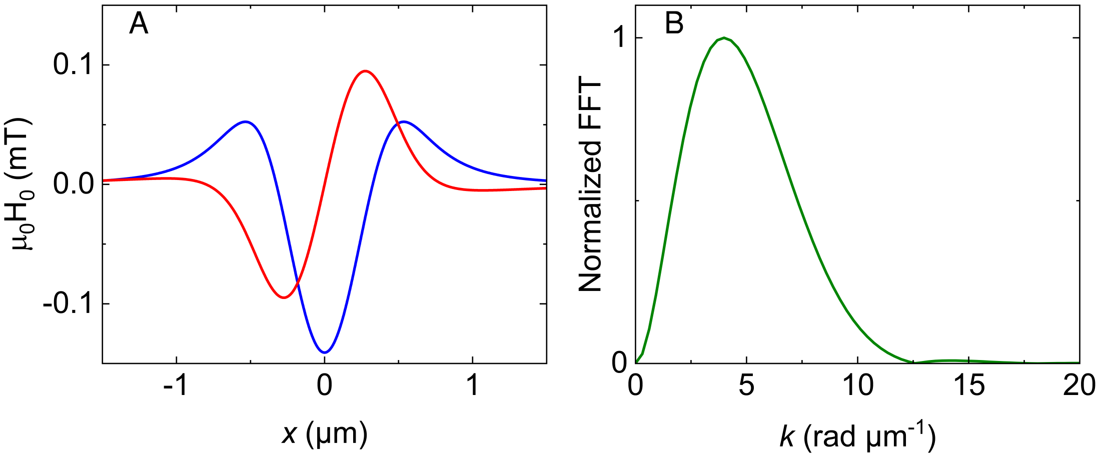

Figure S5: **Ørsted field of the antenna and its normalized Fourier transform.** A Graphs of the x (blue line) and y (red line) components of the Ørsted field of the antenna along the x direction for an alternating current I^1_{AC} = 0.46 mA. B Normalized spatial Fourier transform of the antenna excitation field 350 nm above it with k_{main} = 4 rad µm^{-1}.

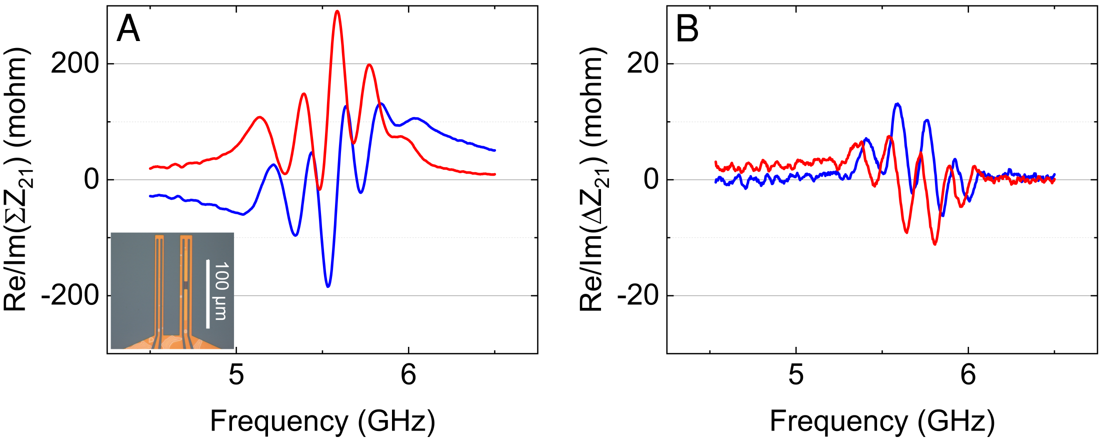

Figure S6: **Inductive signal vs magneto-resistive signal for a 10 µm scale flip-chip device.** A Real part (blue) and imaginary part (red) of the inductive contribution. The inset is an optical microscopy picture of the device. In orange, the exciting antenna (left) and the coplanar waveguide (right) connected to the GMR sensor (gray at the center of the coplanar waveguide). B Real part (blue) and imaginary part (red) of the magneto-resistive contribution.

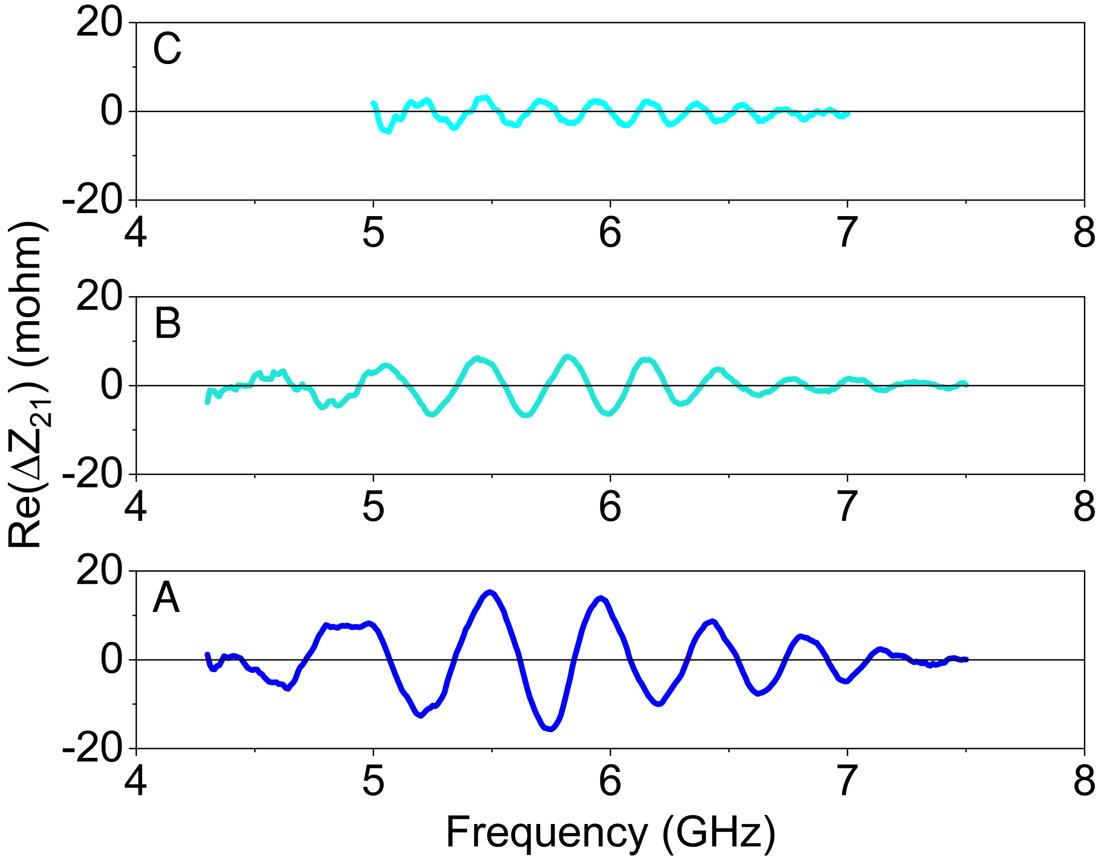

Figure S7: **Decay of the magneto-resistive signal as function of the center-to-center distance between antenna and sensor for a field of 20 mT.** A D =4 µm. B D =6 µm. C D =10 µm.

Caption for Movie S1. Animated view of the simulated device. Visualization of simulated spin-wave and free layer oscillations for a microwave frequency of 6.06 GHz (corresponding to the maximum of the modulus in Fig. 3B) over a full period of oscillation. The color map shows the normalized in-plane component of the dynamic magnetization m_x with the same magnitude of the color scale represented in Fig. 3C. For easier visualization, the equilibrium state of the magnetization has been subtracted and the dynamic magnetization in the GMR stack has been multiplied by a factor of five. Note the interrupted y scale allowing one to visualize both the magnonic waveguide and the GMR stack despite their large vertical separation.

REFERENCES AND NOTES

1. J. Han, P. Zhang, J. T. Hou, S. A. Siddiqui, L. Liu, Mutual control of coherent spin waves and magnetic domain walls in a magnonic device. *Science* **366**, 1121–1125 (2019).

2. Q. Wang, M. Kewenig, M. Schneider, R. Verba, F. Kohl, B. Heinz, M. Geilen, M. Mohseni, B. Lägel, F. Ciubotaru, C. Adelmann, C. Dubs, S. D. Cotofana, O. V. Dobrovolskiy, T. Brächer, P. Pirro, A. V. Chumak, A magnonic directional coupler for integrated magnonic half-adders. *Nat. Electron.* **3**, 765–774 (2020).

3.. Papp, W. Porod, G. Csaba, Nanoscale neural network using non-linear spin-wave interference. *Nat. Commun.* **12**, 6422 (2021).

4. L. Körber, C. Heins, T. Hula, J. V. Kim, S. Thlang, H. Schultheiss, J. Fassbender, K. Schultheiss, Pattern recognition in reciprocal space with a magnon-scattering reservoir. *Nat. Commun.* **14**, 3954 (2023).

5. K. Baumgaertl, D. Grundler, Reversal of nanomagnets by propagating magnons in ferrimagnetic yttrium iron garnet enabling nonvolatile magnon memory. *Nat. Commun.* **14**, 1490 (2023).

6. Y. Fan, M. J. Gross, T. Fakhrul, J. Finley, J. T. Hou, S. Ngo, L. Liu, C. A. Ross, Coherent magnon-induced domain-wall motion in a magnetic insulator channel. *Nat. Nanotechnol.* **18**, 1000–1004 (2023).

7. N. Zenbaa, C. Abert, F. Majcen, M. Kerber, R. O. Serha, S. Knauer, Q. Wang, T. Schrefl, D. Suess, A. V. Chumak, A universal inverse-design magnonic device. *Nat. Electron.* **8**, 106–115 (2025).

8. A. Barman, G. Gubbiotti, S. Ladak, A. O. Adeyeye, M. Krawczyk, J. Gräfe, C. Adelmann, S. Cotofana, A. Naeemi, V. I. Vasyuchka, B. Hillebrands, S. A. Nikitov, H. Yu, D. Grundler, A. V. Sadovnikov, A. A. Grachev, S. E. Sheshukova, J. Y. Duquesne, M. Marangolo, G. Csaba, W. Porod, V. E. Demidov, S. Urazhdin, S. O. Demokritov, E. Albisetti, D. Petti, R. Bertacco, H. Schultheiss, V. V. Kruglyak, V. D. Poimanov, S. Sahoo, J. Sinha, H. Yang, M. Münzenberg, T. Moriyama, S. Mizukami, P. Landeros, R. A. Gallardo, G. Carlotti, J. V. Kim, R. L. Stamps, R.

E. Camley, B. Rana, Y. Otani, W. Yu, T. Yu, G. E. W. Bauer, C. Back, G. S. Uhrig, O. V. Dobrovolskiy, B. Budinska, H. Qin, S. van Dijken, A. V. Chumak, A. Khitun, D. E. Nikonov, I. A. Young, B. W. Zingsem, M. Winklhofer, The 2021 magnonics roadmap. *J. Phys. Condens. Matter* **33**, 413001 (2021).

9. H. Ulrichs, B. Lenk, M. Münzenberg, Magnonic spin-wave modes in CoFeB antidot lattices. *Appl. Phys. Lett.* **97**, 092506 (2010).

10. I. Radu, G. Woltersdorf, M. Kiessling, A. Melnikov, U. Bovensiepen, J. U. Thiele, C. H. Back, Laser-induced magnetization dynamics of lanthanide-doped permalloy thin films. *Phys. Rev. Lett.* **102**, 117201 (2009).

11. H. Qin, R. B. Holländer, L. Flajšman, F. Hermann, R. Dreyer, G. Woltersdorf, S. van Dijken, Nanoscale magnonic Fabry-Pérot resonator for low-loss spin-wave manipulation. *Nat. Commun.* **12**, 2293 (2021).

12. K. Vogt, H. Schultheiss, S. J. Hermsdoerfer, P. Pirro, A. A. Serga, B. Hillebrands, All-optical detection of phase fronts of propagating spin waves in a $Ni_{81}Fe_{19}$ microstripe. *Appl. Phys. Lett.* **95**, 182508 (2009).

13. V. E. Demidov, S. O. Demokritov, B. Hillebrands, M. Laufenberg, P. P. Freitas, Radiation of spin waves by a single micrometer-sized magnetic element. *Appl. Phys. Lett.* **85**, 2866–2868 (2004).

14. T. Sebastian, K. Schultheiss, B. Obry, B. Hillebrands, H. Schultheiss, Micro-focused Brillouin light scattering: Imaging spin waves at the nanoscale. *Front. Phys.* **3**, 35 (2015).

15. V. Vlaminck, M. Bailleul, Spin-wave transduction at the submicrometer scale: Experiment and modeling. *Phys. Rev. B* **81**, 014425 (2010).

16. J. Solano, O. Gladii, P. Kuntz, Y. Henry, D. Halley, M. Bailleul, Spin-wave study of magnetic perpendicular surface anisotropy in single crystalline MgO/Fe/MgO films. *Phys. Rev. Mater.* **6**, 124409 (2022).

17. M. Sushruth, M. Grassi, K. Ait-Oukaci, D. Stoeffler, Y. Henry, D. Lacour, M. Hehn, U. Bhaskar, M. Bailleul, T. Devolder, J. P. Adam, Electrical spectroscopy of forward volume spin waves in perpendicularly magnetized materials. *Phys. Rev. Res.* **2**, 043203 (2020).

18. G. Thiancourt, S. Ngom, N. Bardou, T. Devolder, Unidirectional spin waves measured using propagating-spin-wave spectroscopy. *Phys. Rev. A* **22**, 034040 (2024).

19. T. Devolder, G. Talmelli, S. M. Ngom, F. Ciubotaru, C. Adelmann, C. Chappert, Measuring the dispersion relations of spin wave bands using time-of-flight spectroscopy. *Phys. Rev. B* **103**, 214431 (2021).

20. J. Lucassen, C. F. Schippers, M. A. Verheijen, P. Fritsch, E. J. Geluk, B. Barcones, R. A. Duine, S. Wurmehl, H. J. M. Swagten, B. Koopmans, R. Lavrijsen, Extraction of Dzyaloshinskii-Moriya interaction from propagating spin waves. *Phys. Rev. B* **101**, 064432 (2020).

21. I. Bertelli, J. J. Carmiggelt, T. Yu, B. G. Simon, C. C. Pothoven, G. E. W. Bauer, Y. M. Blanter, J. Aarts, T. van der Sar, Magnetic resonance imaging of spin-wave transport and interference in a magnetic insulator. *Sci. Adv.* **6**, eabd3556 (2020).

22. B. G. Simon, S. Kurdi, J. J. Carmiggelt, M. Borst, A. J. Katan, T. van der Sar, Filtering and imaging of frequency-degenerate spin waves using nanopositioning of a single-spin sensor. *Nano Lett.* **22**, 9198–9204 (2022).

23. S. Wintz, V. Tiberkevich, M. Weigand, J. Raabe, J. Lindner, A. Erbe, A. Slavin, J. Fassbender, Magnetic vortex cores as tunable spin-wave emitters. *Nat. Nanotechnol.* **11**, 948–953 (2016).

24. N. Brookes, D. Betto, K. Cao, Y. Lu, K. Kummer, F. Giustino, Spin waves in metallic iron and nickel measured by soft x-ray resonant inelastic scattering. *Phys. Rev. B* **102**, 064412 (2020).

25. M. N. Baibich, J. M. Broto, A. Fert, F. Nguyen van Dau, F. Petroff, P. Etienne, G. Creuzet, A. Friederich, J. Chazelas, Giant magnetoresistance of (001)Fe/(001)Cr magnetic superlattices. *Phys. Rev. Lett.* **61**, 2472–2475 (1988).

26. G. Binasch, P. Grünberg, F. Saurenbach, W. Zinn, Enhanced magnetoresistance in layered magnetic structures with antiferromagnetic interlayer exchange. *Phys. Rev. B* **39**, 4828 (1989).

27. B. Dieny, Giant magnetoresistance in spin-valve multilayers. *J. Magn. Magn. Mater.* **136**, 335–359 (1994).

28. J. Moulin, “Microscopie magnétique locale par intégration de nanocapteurs magnétorésistifs,” thesis, Université Paris-Saclay (2020). [Local magnetic microscopy using integrated magnetoresistive nanosensors].

29. J. Moulin, A. Doll, E. Paul, M. Pannetier-Lecoeur, C. Fermon, N. Sergeeva-Chollet, A. Solignac, Optimizing magnetoresistive sensor signal-to-noise via pinning field tuning. *Appl. Phys. Lett.* **115**, 122406 (2019).

30. J. R. Childress, R. E. Fontana Jr., Magnetic recording read head sensor technology. *C. R. Phys.* **6**, 997–1012 (2005).

31. C. Fermon, M. Van de Voorde, *Nanomagnetism: Applications and Perspectives* (John Wiley & Sons, 2016).

32. M. Pannetier, C. Fermon, G. Le Goff, J. Simola, E. Kerr, Femtotesla magnetic field measurement with magnetoresistive sensors. *Science* **304**, 1648–1650 (2004).

33. B. Dieny, I. L. Prejbeanu, K. Garello, P. Gambardella, P. Freitas, R. Lehndorff, W. Raberg, U. Ebels, S. O. Demokritov, J. Akerman, A. Deac, P. Pirro, C. Adelmann, A. Anane, A. V. Chumak, A. Hirohata, S. Mangin, S. O. Valenzuela, M. C. Onbaşlı, M. d’Aquino, G. Prenat, G. Finocchio, L. Lopez-Diaz, R. Chantrell, O. Chubykalo-Fesenko, P. Bortolotti, Opportunities and challenges for spintronics in the microelectronics industry. *Nat. Electron.* **3**, 446–459 (2020).

34. R. W. Damon, J. Eshbach, Magnetostatic modes of a ferromagnet slab. *J. Phys. Chem. Solid* **19**, 308–320 (1961).

35. C. Chopin, J. Torrejon, A. Solignac, C. Fermon, P. Jendritza, P. Fries, M. Pannetier-Lecoeur, Magnetoresistive sensor in two-dimension on a 25 μm thick silicon substrate for in vivo neuronal measurements. *ACS Sens.* **5**, 3493–3500 (2020).

36. J. Torrejon, A. Solignac, C. Chopin, J. Moulin, A. Doll, E. Paul, C. Fermon, M. Pannetier-Lecoeur, Multiple giant-magnetoresistance sensors controlled by additive dipolar coupling. *Phys. Rev. Appl.* **13**, 034031 (2020).

37. O. Gladii, "Spin wave propagation and its modification by an electrical current in Py/Al_2O_3, Py/Pt and Fe/MgO films," thesis, Université de Strasbourg (2016).

38. M. Haidar, "Role of surfaces in magnetization dynamics and spin polarized transport: A spin wave study," thesis, Universite de Strasbourg (2012).

39. M. P. Grassi, "Spin waves in inhomogeneous magnetization distributions," thesis, Université de Strasbourg (2021).

40. B. Kalinikos, A. Slavin, Theory of dipole-exchange spin wave spectrum for ferromagnetic films with mixed exchange boundary conditions. *J. Phys. C Solid State Phys.* **19**, 7013–7033 (1986).

41. C. Kittel, On the theory of ferromagnetic resonance absorption. *Phys. Rev.* **73**, 155–161 (1948).

42. M. Buchner, K. Höfler, B. Henne, V. Ney, A. Ney, Tutorial: Basic principles, limits of detection, and pitfalls of highly sensitive SQUID magnetometry for nanomagnetism and spintronics. *J. Appl. Phys.* **124**, 161101 (2018).

43. T. Bracher, "Parallel parametric amplification of spin waves in micro-structures," thesis, Fachbereich Physik der Technischen Universitat Kaiserslautern (2015).

44. A. Vansteenkiste, J. Leliaert, M. Dvornik, M. Helsen, F. Garcia-Sanchez, B. van Waeyenberge, The design and verification of MuMax3. *AIP Adv.* **4**, 107133 (2014).

45. Y. Henry, O. Gladii, M. Bailleul, Propagating spin-wave normal modes: A dynamic matrix approach using plane-wave demagnetizating tensors. arXiv:1611.06153 [cond-mat.mes-hall] (2016).

46. J. Robert, P. Turek, M. Bailleul, A. K. Boudalis, Broadband electron paramagnetic resonance of a molecular spin triangle. *Phys. Chem. Chem. Phys.* **23**, 20268–20274 (2021).
